# Reelin’ In The Years: Age and Selective Restriction of Liberty in the COVID-19 Pandemic

**DOI:** 10.1007/s11673-023-10318-8

**Published:** 2023-12-04

**Authors:** David Motorniak, Julian Savulescu, Alberto Giubilini

**Affiliations:** 1 Faculty of Medicine, Nursing and Health Sciences, Monash University, Clayton, Australia; 2 Yong Loo Lin School of Medicine, National University of Singapore, Singapore, Singapore; 3 Oxford Uehiro Centre for Practical Ethics and Wellcome Centre for Ethics and Humanities, University of Oxford, Oxford, UK; 4 Murdoch Children’s Research Institute, Melbourne, Australia

**Keywords:** Selective lockdown, Focused-protection strategies, COVID-19, Freedom, Age discrimination

## Abstract

During the COVID-19 pandemic, focused protection strategies including selective lockdowns of the elderly were proposed as alternatives to general lockdowns. These selective restrictions would consist of isolating only those most at risk of COVID-19 hospitalization and subsequent use of healthcare resources. The proposal seems to have troubling implications, including the permissibility of selective lockdown on the basis of characteristics such as ethnicity, sex, disability, or BMI. Like age, these factors also correlated with an increased risk of hospitalization from COVID-19. In this paper, we argue that age has meaningful differences as a morally relevant characteristic in the justification for selective restrictions of liberty. Thus, it might justify selective freedom restrictions in a way in which other factors might not. We offer four moral domains that separate age from other proxies: empiricism, operationality, discrimination, and disparity.

## Introduction

The pandemic experience has raised the question of whether and to what extent freedom restrictions can be applied unequally during future public health emergencies.

Despite lockdown stemming the tide of initial COVID-19 deaths, lockdown measures imposed a considerable cost on society. The COVID-19 virus inflicted a significant cost in terms of lives lost. Lockdown measures resulted in substantial job losses, a global recession, and deteriorated physical and mental health, causing substantial death and disability (Rogers and Cruickshank 2021; Banks, Karjalainen, and Propper 2020). For the purposes of this article, “*lockdown”* refers to mandatory stay-at-home orders and subsequent closure of businesses. Legal mandates were introduced given anticipated challenges in ensuring continued effectiveness and widespread compliance in an ultimately enduring emergency (Murphy, et al. 2020).

In 2020, Savulescu and Cameron argued for selective lockdown of the elderly, avoiding the need for a population-wide lockdown by isolating only those most at risk of COVID-19 hospitalisation and subsequent use of healthcare resources. This solution would allow social and economic activities to continue while offering a significant degree of protection to the most vulnerable groups (Williams, et al. 2021). Lockdown measures and their hitherto unquantified societal, psychological, and economic costs would be largely avoided.

Other authors have argued that this *age-based lockdown* may set a precedent for discrimination in future public health emergencies (Blunkett 2020). Taking selective lockdown of the elderly to its natural endpoint provokes discussion about selective lockdown of those with other morally relevant characteristics. In particular, individuals of certain ethnicities, sex, disability, immune-status, or Body Mass Index (BMI) also have an increased risk of hospitalization from COVID-19 and presumably hitherto unknown infectious diseases (Humberstone 2020).

If COVID-related risk profile is considered the relevant criterion for targeting population groups in selective restrictions, the liberty of which groups should be restricted? That is, *assuming* restriction of liberty is justified in the COVID-19 context (an assumption we will not discuss here), who—if anyone—should be coerced?

Savulescu and Cameron (2020) rejected criticisms of ageism, understood as unfair discrimination on the basis of age. That is because, they argued, discrimination is only unfair when it is based on arbitrary, morally irrelevant traits and contributes to making someone worse off than others. But increased risk of disease and pressure on limited health resources are morally relevant characteristics that can, in certain circumstances, make age-based discrimination not unfair. The possible implication that we should also restrict people’s freedom on the basis of their ethnicity, sex, or other problematic characteristics has not been properly addressed. One worry is that this policy might result in racist, sexist, or otherwise ethically problematic forms of discrimination. In this paper, we argue that age has meaningful differences as a morally relevant characteristic in restricting liberty.

In light of the ensuing discussion about whose freedom to limit, and the risk of unfair discrimination that comes with treating different groups unequally, there is a question to be asked as to whether and when targeted public health restrictions might open the door to unfair discrimination and more specifically to ageism.

Addressing all the nuances that define ageism and make it ethically and socially problematic (Levy and Macdonald 2016) would require a separate discussion that is beyond the scope of this paper. For example, there is a discussion as to what counts as *old* age (Giles and Reid 2005). However, we will discuss features of differential treatment that would make a policy ageist, and we will argue that they do not apply to age-based restrictions in the case of a pandemic like COVID-19.

We offer four moral domains that separate age from other proxies and which we will call, respectively, empiricism, operationality, discrimination, and disparity. Lockdown measures, although increasingly irrelevant for the now endemic COVID-19 pandemic, may be brought back to the table by policymakers to tackle potential future pandemics if healthcare systems are placed at risk. It can be debated whether protecting healthcare systems should be a priority of government response to pandemics or other public health crises; however, as a matter of fact, this often was presented as the rationale behind tight restrictions, which suggests that it will likely be considered a priority in future public health emergencies. As we move beyond COVID-19, restrictions for public health purposes will remain—rightly or wrongly—an option on the table of policymakers. And with it, the ethical issue of fairly applying freedom restrictions across a population will arise.

Previous authors rightfully draw attention to increased elder abuse, nursing neglect, and suicide rates among the elderly during lockdown, which may contrast the perceived benefits for the elderly of age-based restrictions in terms of protection from the virus (Giri, Chenn, and Romero-Ortuno 2021; Yunus, Abdullah, and Firdaus 2021; Sarangi, Fares, and Eskander 2021). These challenges are valid, but they are reasons against lockdowns in general, not against selecting upon age itself. These problems are not necessarily worsened by age-based lockdown. In this article we will not provide arguments for or against freedom restrictions in general. Instead, we will argue that if freedom restrictions in the form of lockdowns are (rightly or wrongly) adopted, age-based lockdowns are not unfairly discriminatory when tackling a virus like COVID-19 that threatens the elderly far more than it threatens the young.

## Just and Unjust Discrimination

As Grill and Dawson (2017) propose, targeted measures in public health policy increase the extent and likelihood of realising public health goals. Discriminating means acting on the basis of differences between individuals. It can be harmful and unfair in certain cases but beneficial and fair in others. As Cameron, et al. outline, sometimes discrimination on morally relevant differences “is not just permissible, but is necessary to achieve equitable outcomes” (Cameron, et al. 2021).

In the case of COVID-19, not everyone was at equal risk of requiring hospitalization if infected, and not every population group posed an equal risk of burdening healthcare systems. The elderly present a drastically higher risk of hospitalization from COVID-19 than their younger counterparts (Cameron, et al. 2021). Indeed, an age-based lockdown would still fulfil the justification for conventional lockdowns by flattening the curve not so much of infections in the population, but of hospital admissions, which are largely constituted by older adults. Spread of the virus may intensify among younger, less vulnerable groups who are at very low risk of being hospitalized and harming healthcare capacity, but the curve of hospitalizations would remain low.

An age-based lockdown would not be merely “arbitrary incarceration,” as some have called it (Hill 2020). There is at least one morally relevant difference—risk of harm posed—that makes selective lockdown non-arbitrary and could justify differential treatment of the elderly. There is a separate question as to what level of risk of harm to others would justify non-consensual lockdown, but this is an issue raised both by population-wide and age-based lockdown, and we will not address it here. The question we will address is whether selective, aged-based lockdowns are discriminatory, compared to population-wide lockdowns.

In fact, we already accept that discrimination based on age can be not unfair, including in the pandemic context. For instance, the elderly have been prioritized in access to COVID-19 vaccines, when these were scarce. Age-based vaccine distribution has been embraced as a method of justly distributing benefits and harms. The elderly are more vulnerable, and vulnerability is a morally relevant difference.

The problem is that, as we said above, sex (male), race, disability, and BMI (and curiously, political membership) also have a bearing on COVID-19 risk and consequently the risk one poses to society’s limited resources (Humberstone 2020; Porteny, et al. 2022). However, as Savulescu and Cameron outline, it is one thing to recognize a relevant difference and the benefit that may be obtained from discriminating; it is another to justify a discriminatory policy that achieves this benefit. So why does age stand out compared to other risk factors?

## The Empirical Argument

### The Elderly have the Most Risk

COVID-19 patients aged 65 and over suffered mortality rates 62 times higher than their younger counterparts and 81 per cent of COVID-19 hospitalizations were made by individuals aged over 65 (Yanez, et al. 2020; Garg, et al. 2020). If we state that the goal of restrictions of liberty during a pandemic is to limit lives lost and to maintain day-to-day life and functioning healthcare systems, then targeting those that present the greatest risk to society’s collective resources is the most justifiable option: it would diminish the burden on healthcare systems while preserving at least some degree of day-to-day life and level of freedom across society, even if unequally distributed.

No other demographic reaches parity with the degree of indirect risk posed by those over 65-years-old, both in COVID-19 and most other infectious diseases. There is a predominance for admission among males with COVID-19, but only 5 per cent more than their female counterparts (Savulescu and Cameron 2020). African-Americans with COVID-19, disproportionately represented among hospital admissions, appear at a rate only approximately twice that of their white counterparts (Centre for Disease Control and Prevention 2022). Age is a stronger risk factor.

O’Hanlon argues that age is not, in fact, an effective proxy for COVID-19 risk (O’Hanlon 2020). We agree that there are many robust and healthy individuals over the age of 70-years-old and even 80-years-old. It might be claimed that there are more accurate proxies of risk available—an amalgamation of comorbidities, polypharmacy, BMI, lung function, and immune status perhaps. However, as we point out in the *Operationality* section, it is unlikely to be feasible to construct, identify, and enforce restrictions upon such a group.

Public health policy necessarily ignores a degree of diversity in individual risk profiles and relevant personal circumstances when dealing with the collective good. Any construction of a group necessarily ignores at least some relevant individual characteristics. We are not arguing that the risk of severe COVID-19 is universal among the elderly, which it certainly is not; however, the correlation between older age and susceptibility is clear and strong.

### The Most Elderly have the Most Benefit

Given that being confined provides considerable protection against the virus, a selective restriction of liberty will benefit the elderly most in minimizing COVID risks.

Yet it is not enough that selective restriction of liberty benefits older individuals most—the level of coercion must also be proportionate to the benefit. Since it was judged to be proportionate to restrict younger people for much smaller individual benefits during the pandemic, it must be judged proportionate to coerce the elderly—since they have the most to gain from the restrictions. Granted, the premise of this argument is questionable: many would argue that it was not proportionate to restrict younger people. But that would be a reason against lockdown *tout court*, rather than against selective lockdowns of the elderly.

While many among the elderly are socially isolated and would experience psychological harm from lockdown, this harm would be inevitable in a generalized lockdown. Again, this would be a reason against lockdown *tout court*. If we accept that restrictions are justified in the first place—which we do for the sake of argument—the answer to the problem of psychological harm consists of taking more care of the well-being of the vulnerable and the isolated, including during lockdown. In fact, this is something we should have done better for everyone, including children, during lockdown. The fact that the harm would be imposed selectively is not relevant to the moral issue at stake, that is, the badness of psychological harm. It is just a reason to do more to prevent it once we have accepted the legitimacy of restrictions. Even if these harms were disproportionately severe among the elderly, they would not necessarily be made worse by an age-based lockdown and in fact, may be made more amenable to remedy, as we also argue in the "Empirical challenges" section.

## The Operationality Argument

As we have pointed out, there are operational (or *feasibility*) considerations that must be contemplated in selecting who will be coerced. In considering these logistics, it is necessary to consider if 1) cohorts of individuals can be reasonably identified and delineated, 2) lockdown of this cohort is feasible, and 3) lockdown of this cohort will effectively limit disease burden.

First, can all traits reasonably be identified for our purposes? For example, what if race was identified to be a more accurate predictor for COVID-19 hospitalization? Assume that South Asian individuals held the highest risk for COVID-19 morbidity. Our argument would imply that the South Asian population should enter lockdown to prevent limited healthcare resources from being strained, serving society at large. However, while age is an “epistemically robust category,” many traits do not occupy discrete classifications (John 2020).

Age is quantifiable. We can say that Daniel is verifiably 24-years-old, Jason 58, Alex 41, etc. While race or disability may be quantifiable to a degree, their blurred classification and the role of self-identification prevents utilization as a method of accurately measuring COVID-19 morbidity. For example, when would someone be South Asian *enough* to enter lockdown? This argument will not extend to all risk factors—for example BMI can be given a number. If sufficiently predictive of hospitalization or death, it could be treated like age. Similarly, sex is a firm biological category. But there are other operational conditions that rule out sex as a good criterion for selective lockdowns.

The second condition to justify selection is the feasibility of a lockdown of a certain cohort from active society. Selective lockdown of an entire sex is not feasible. Mandatory lockdown of an entire sex would require removal of 50 per cent of the population. Given that the intention of a selective lockdown was to preserve at least some social and economic activity, this alternative would be unrealistic. Instead, demographics over 65-years-old make up some 15 per cent of the population, are largely not employed, and when employed, operate in areas characterized by low productivity (Giammetti, et al. 2022). Isolating this cohort without significant economic disruption, whether for COVID-19 or other diseases, seems feasible.

For our third condition, we consider if lockdown of the chosen cohort will effectively limit disease burden. In many countries, the ethnic minorities at highest risk from COVID-19 typically make up a small fraction of a given population. The same argument may be made for organ recipients or profoundly immunosuppressed patients, who indeed suffer the greatest COVID-related mortality and can be readily identified (note that those at greatest risk are likely already voluntarily isolating in the absence of coercive policy) (Myerson, et al. 2021). If only a small fraction of people are isolated, even if this is the most vulnerable group known, there will still remain enough vulnerable individuals in society to strain limited healthcare resources. A sufficiently large *and* vulnerable demographic must be selected such that enough disease burden is withdrawn, preventing limited healthcare resources from being consumed. This is, after all, the goal of our selective policy.

What about our previous example in *The Empirical Argument*, of an amalgamated group of risk factors, thus necessitating a lesser restriction of liberty? Certainly, this must be possible in developed nations using data from electronic medical records; over fifty predictive analyses were constructed during COVID-19 (Wynants, et al. 2020). However, constructing and utilizing accurate proxies presents unique challenges for authorities. For example, obtaining unanimous consent to access and link private medical records is unpalatable and impractical. There is a problem with the feasibility of “cleaning” massive volumes of data, and the retrospective collection of data offers no guarantee that an individual currently has some disease or medication (and who may very well be incentivized to argue that they no longer do in the interest of their liberty). We find that such a solution, while a gold standard, would challenge the first of our outlined conditions: that risk could be reasonably identified. Indeed, many of the models developed were labelled in systematic review as “poorly reported [and] highly biased,” broadly reporting age as the most important feature in predicting mortality (Wynants, et al. 2020).

We acknowledge that there are other dimensions to feasibility. The elderly, like anyone, will necessarily have interactions with those not isolating (say, grocery, medical appointments, shared living and so on). We acknowledge these are problematic aspects and confront them later in this article.

## The Harmful Discrimination Argument

There are reasons to be cautious of a selective restriction of liberty. Discrimination, however justified, may provoke prejudice. We are arguing here that discrimination on the basis of age is justified when certain conditions are met. But someone may be empowered by this precedent to unjustly justify prejudice and antagonism of the elderly. There are fears that justified discrimination might open the door to the unfair type, namely ageism.

It is unclear how serious this risk would be, though. First, age is less *socially charged* or linked to social disadvantage than its comparators of ethnicity or sex and less likely to promote the harmful discrimination we intend to avoid. Comparatively, the use of age as a corollary for COVID-19 severity is intuitive in the public’s eyes given the visibility of older individuals in the COVID-19 narrative; it is unlikely to be affected by external biases. Importantly, “this category, [age], is understandable” by the public in its relation to risk (John 2020). And second, we all get older—age is an inevitable variable that will apply to the entire population, bar premature death. On the contrary, not everyone will be of a particular race, sex, or disability. Moreover, each actor would have a chance throughout their lifetime of being placed under such a restriction, assuming the risks posed by COVID-19 or similar public health threats remain present.

Even if the potential for unfair discrimination argument applies to race (or other traits) and constitutes a strong reason against race-based selective lockdowns, it does not constitute an equally strong reason against age-based lockdown.

However, we do not mean to diminish the importance of recognizing ageism, which has significant connotations for one’s physical and mental health, including decreased life expectancy (Kessler and Bowen 2020; Australian Human Rights Commission 2020). We should combat ageism regardless of the presence of selective measures. And we certainly do have the power to respect and praise the elderly positively in public health messaging.

## The Disparity Argument

Initially, a general lockdown appears egalitarian as individuals share the same limitations on movement and action. However, while equal in implementation, the same cannot be said for outcomes.

The young and old often have competing interests. Giubilini (2021) writes that the costs of population-wide lockdowns accrue mostly in younger demographics who have a lot to lose and little to benefit from lockdowns, while the benefits accumulate mostly among the elderly—chiefly, less transmission and subsequent mortality. This seems to make population-wide lockdowns ageist against the young. Selecting on the basis of age corrects this disparity; however, selecting on other traits would not have the value of correcting disparities.

What do we mean by *harms* to the young? They are considerable. COVID-19-related government economic intervention produced $24 trillion in debt by 2021, a cost that will largely be borne by future generations (Pesek 2021). Industries devastated by lockdown such as hospitality and retail were dominated by younger cohorts (Gilfillan 2020). Young people beginning their academic and professional lives faced immense disruption with university closures. Mental illness was disproportionately exacerbated in this group throughout lockdown (Bhavsar, et al. 2021; Gao, Bagheri, and Furuya-Kanamori 2022). Young people have endured delayed access to vaccines, preventing re-entry to a society that (previously) demanded vaccination passports for reintegration.

Many of these harms also apply to older individuals; they are not immune. However, older cohorts typically have greater financial well-being and are more resilient to financial shocks (Collins and Urban 2019). We certainly acknowledge poverty and frequent inadequacy of pensions among this population, which worsened during COVID-19. However, while there are adverse impacts of lockdown, they are not uniquely of age-based lockdown. Population-wide lockdowns (the alternative against which we are assessing age-based lockdowns) would present the same problems, likely to a higher degree. Indeed, many of the financial stresses identified by this cohort (i.e., supporting adult children, volatile markets) would surely be alleviated by an age-based lockdown (Jhuremalani, et al. 2022). Lockdown-related harms to the elderly could in fact be mitigated by an age-based lockdown where increased freedom among other cohorts would improve nursing care and other services, with flow-on effects for the well-being of the elderly. And what about unequal *benefits*? Minimizing transmission of an infectious disease benefits everyone. However, given that older demographics are far more likely to suffer the severe sequelae of the disease, minimized transmission is of more significant benefit to the elderly than their younger counterparts. Conspicuously, given overall increased utilization of healthcare resources by older individuals, preserved healthcare capacity would also be of significant benefit to those restricted.

Adopting an age-based lockdown corrects inequalities present in lockdowns. It does not redistribute harms from the young to the old, but relieves greater pressure placed on the young, while also benefitting the old. Conversely, a hypothetical race-based lockdown (or any other) would not correct inequalities but rather reinforce social disadvantages.

## Further Considerations

### Empirical Challenges

We have assumed that the elderly can be completely isolated from COVID-19 transmission, as assumed in past modelling (Ragonnet, et al. 2020). Complete isolation is unlikely to be feasible, with basic activities, intergenerational households, and residential aged-care facilities necessitating some exposure. This contact, even mitigated, may challenge the effectiveness of our proposal. Nursing homes, for instance observed some of the highest rates of COVID-19 mortality (Giri, Chenn, and Romero-Ortuno 2021).

However, it is important to remember once again that population-wide lockdown is the baseline against which we are here assessing selective lockdowns, and it is not clear how the former, which many countries did implement, is more feasible than the latter. If anything, it seems more feasible to provide for the needs of the elderly (including those in nursing homes) during a selective lockdown, where the rest of society can find ways to assist the isolated elderly, than during a full lockdown, where everyone else is also confined. Indeed, if liberties are to be so constrained as to not cause harm to the elderly, relaxation of any future lockdown may never be justified.

It seems that those raising the issue of feasibility in favour of full lockdown are comparing selective lockdown to a situation without lockdown (Lawrence and Harris 2021; Kao 2021). But that is inconsistent with their view, as it would require them to drop their support for general lockdown. If you endorse general lockdown as the more ethical alternative to selective lockdown, you would need to use general lockdown as the baseline against which to assess pros and cons of the selective one.

### Limited Life Expectancy

Lawrence and Harris outline what they perceive as “pick[ing] on the elderly” (Kao 2021). The authors identify a “weakness of the elderly” that is “proportionally greater than that experienced by most other age groups”: reduced life expectancy. It is argued that an individual with “only 1 or 2 years to live” suffers a “relatively greater loss” than a comparable person with many years remaining (Kao 2021). Consequently, older demographics have more right to freedom from restrictions. This argument faces several problems.

First, if the alternative to selective lockdown is a full lockdown, then this argument doesn’t seem very strong as the elderly would also lose their liberty in a full lockdown.

Second, this argument fails to consider the value of liberty to the young. The relative youth of an individual is not a valid reason to devalue the importance of their liberty. As we illustrated previously, there are reasons to consider the loss of liberty to the young particularly detrimental to their development and future success.

Third, it fails to consider why the elderly have less life expectancy—–because they have already lived more. The fact that a cohort is at the other end of their journey through life does not grant a greater claim to freedom, especially if they have already experienced such freedoms. Ideally, everyone should keep their own freedom, but if we assume that someone’s freedom is to be restricted, having enjoyed more of it so far cannot count against restricting one’s freedom.

## Conclusion

Selective lockdown on the basis of age can be ethically justified. One may claim this policy is harsh in restricting the liberty of the elderly. However, it can be no more harsh than a general lockdown that would restrict everyone’s liberty.

Unlike selection of other morally relevant traits, selection on the basis of age enjoys greater empirical support, can be operationalized, and is dissimilar in salient ethical points from other kinds of differential treatments involving risks of unfair discrimination.

As we move beyond COVID-19, we should consider what characteristics are selected when implementing future coercive policies not only to protect healthcare systems but also to protect liberties and allow important social and economic activities to continue.
